# COLOR III: a multicentre randomised clinical trial comparing transanal TME versus laparoscopic TME for mid and low rectal cancer

**DOI:** 10.1007/s00464-015-4615-x

**Published:** 2015-11-04

**Authors:** Charlotte L. Deijen, Simone Velthuis, Alice Tsai, Stella Mavroveli, Elly S. M. de Lange-de Klerk, Colin Sietses, Jurriaan B. Tuynman, Antonio M. Lacy, George B. Hanna, H. Jaap Bonjer

**Affiliations:** Department of Surgery, VU University Medical Centre, Postbus 7057, 1007 MB Amsterdam, The Netherlands; Department of Surgery, Gelderse Vallei Hospital Ede, Ede, The Netherlands; Department of Surgery and Cancer, Imperial College London, London, UK; Department of Surgery, Hospital Clinic de Barcelona, Barcelona, Spain

**Keywords:** Transanal, Total mesorectal excision, TME, TaTME, Laparoscopic, Rectal cancer, Surgical quality

## Abstract

**Introduction:**

Total mesorectal excision (TME) is an essential component of surgical management of rectal cancer. Both open and laparoscopic TME have been proven to be oncologically safe. However, it remains a challenge to achieve complete TME with clear circumferential resections margin (CRM) with the conventional transabdominal approach, particularly in mid and low rectal tumours. Transanal TME (TaTME) was developed to improve oncological and functional outcomes of patients with mid and low rectal cancer.

**Methods:**

An international, multicentre, superiority, randomised trial was designed to compare TaTME and conventional laparoscopic TME as the surgical treatment of mid and low rectal carcinomas. The primary endpoint is involved CRM. Secondary endpoints include completeness of mesorectum, residual mesorectum, morbidity and mortality, local recurrence, disease-free and overall survival, percentage of sphincter-saving procedures, functional outcome and quality of life. A Quality Assurance Protocol including centralised MRI review, histopathology re-evaluation, standardisation of surgical techniques, and monitoring and assessment of surgical quality will be conducted.

**Discussion:**

The difference in involvement of CRM between the two treatment strategies is thought to be in favour of the TaTME. TaTME is therefore expected to be superior to laparoscopic TME in terms of oncological outcomes in case of mid and low rectal carcinomas.

Annually approximately 737.000 patients are diagnosed with rectal cancer worldwide [1]. The standard potentially curative treatment of rectal cancer is total mesorectal excision (TME). With the introduction of laparoscopic TME, concerns arose about the oncological safety. The COlorectal cancer Laparoscopic or Open Resection (COLOR) II trial demonstrated improved short-term outcomes and similar long-term outcomes after laparoscopic resection of rectal cancer, compared with open resection [2, 3].

However, particularly resection of mid and low rectal cancer is technically demanding due to tapering of the mesorectum in the pelvis and the forward angle of the distal rectum rendering this part of the rectum less accessible from the abdominal cavity. These factors predispose to incomplete mesorectal excision and involved circumferential resection margins (CRMs), with consequent local recurrences. Moreover, high morbidity rates are reported as result of poor anastomotic techniques and high conversion rates because of the limited view on the distal margin of the tumour and difficult mobilisation in the narrow pelvis. Despite the increasing uptake of laparoscopic TME in the treatment of rectal cancer, conversion rates to open procedures are reported up to 34 % [2, 4, 5]. Conversion is frequently needed in male, obese patients or in case of bulky or distally located tumours. Furthermore, mid and low rectal cancer surgery is associated with poor functional outcome with high colostomy rates compared to high rectal cancer [2]. Large randomised trials reported rates of abdominoperineal resection (APR) in laparoscopic rectal cancer resection of 25–29 % [2, 4].

To improve visualisation and potentially improve functional and oncological results, the transabdominal transanal (TATA) technique was introduced in the 1990’s, which included an open approach from below to rectal tumours located in the distal one-third of the rectum [6]. The use of single port laparoscopic platforms has enabled the introduction of transanal TME (TaTME) by Lacy in 2010. Both techniques have in common to adhere to TME principles, achieving tumour-free distal and circumferential margins (CRMs) and harvesting a minimum of 12 lymph nodes for pathological assessment [7]. The TaTME technique takes the most important developments in rectal cancer surgery from the last 30 years and combines them into one surgical technique [8].

TaTME for mid and low rectal cancer has potential benefits: better specimen quality with better radicality, less morbidity as result of better anastomotic techniques and less conversions and more sphincter-saving rectal resections without compromising oncological outcomes.

Several groups have already demonstrated that TaTME can be performed safely with a promising amount of intact specimens and low rates of involved CRM [9–14]. The next crucial step of assessing a surgical innovation is a randomised controlled trial. Therefore, a randomised trial is needed to evaluate the role of TaTME in rectal cancer and to assess oncological outcomes. The COLOR III trial has been designed to compare short- and long-term outcomes of transanal and laparoscopic TME for mid and low rectal cancer.

## Patients and methods

COLOR III trial is an international, multicentre, superiority, randomised trial comparing TaTME and laparoscopic TME as the surgical treatment of mid and low rectal carcinomas.

### Eligibility

A total of 1098 consecutive patients scheduled for resection of a solitary mid or low rectal carcinoma (5–10 and 0–5 cm from anal verge on MRI) will be included in the COLOR III trial. Patients with stage I–III rectal cancer for whom TME is indicated, suitable for elective resection, with a rectal carcinoma observed at colonoscopy and histologically proven through biopsy are eligible. The distal border of the tumour has to be within 10 cm of the anal verge on MRI scan. A CT scan of the thorax and abdomen should be performed to exclude distant metastases. Patients after neoadjuvant therapy, patients with any BMI, as well as patients with previous abdominal or pelvic surgery can be included. Furthermore, patients with tumours that are downstaged can be included. This means patients with a distance of <2 mm between tumour and mesorectal fascia, tumour ingrowth in the anal sphincter complex or m. levator ani or T4 tumours having: distance more than 2 mm between tumour and mesorectal fascia, no ingrowth or no T4 tumour after neoadjuvant therapy can be included. Informed consent will be obtained from all eligible patients in accordance with the requirements of the local ethical board.

Exclusion criteria are T1 tumours which can be treated by local excision, T3 tumours with margins <1 mm to the endopelvic fascia, tumours with ingrowth in the internal sphincter or m. levator ani and all T4 tumours as staged through MRI scan prior to neoadjuvant therapy. Other causes for exclusion are previous rectal surgery, pregnancy, age <18 years, absolute contraindications to general anaesthesia or prolonged pneumoperitoneum (ASA score of more than III), signs of acute intestinal obstruction or synchronous abdominal surgery. Furthermore, a medical history of familial adenomatous polyposis coli, hereditary non-polyposis colorectal cancer, active Crohn’s disease or colitis ulcerosa or other malignancies, except adequately treated basocellular skin carcinoma or in situ cervix uteri carcinoma, will result in exclusion.

All participating centres in the COLOR III trial will keep the coordinating centre informed of all patients presenting with rectal cancer. Data on patients with rectal cancer who are not included in the COLOR III trial will be registered.

### Randomisation

Once eligibility has been established, patient details have been noted and the preoperative MRI is centrally reviewed, patients will be allocated to either transanal or laparoscopic TME. Randomisation will be performed by computer through the internet and will be stratified for preoperative (chemo)radiotherapy, T-stage, height of the tumour (mid or low) and gender. Patients will be randomised in a 2:1 ratio, in favour of the TaTME. Data will be analysed on ‘intention to treat’ basis in case patients are not subjected to the randomised treatment modality.

### Surgical procedure

Included surgical procedures to obtain TME are: 1. low anterior resection (LAR) with colorectal anastomosis, 2. LAR with coloanal anastomosis and 3. intersphincteric abdominoperineal resection (APR).

Excluded is an extralevator abdominoperineal excision (ELAP) (indicated in patients with tumour ingrowth in the anal sphincter complex or m. levator ani).

Complete laparoscopic excision of the total mesorectum is mandatory to qualify the procedure as a ‘laparoscopic TME’. The level of transection of the inferior mesenteric artery is up to the surgeon’s preference. Both right and left hypogastric nerves should be preserved. The splenic flexure should be mobilised when undue tension at the anastomosis is likely. Other aspects of the surgical procedure such as type of anastomosis, use of diverting ileostomy and drainage of surgical field are up to the discretion of the surgeon.

In TaTME, the rectum is being mobilised transanally according to TME principles. TaTME is defined as dissection of the distal one-third of the mesorectum. After resection of the rectum and the mesorectum, a hand sewed or stapled anastomosis is created according to the preference of the performing surgeon, as well as creation of a diversion ileostomy and drainage of the surgical field.

In both treatment arms, the use of single port as well as multiport laparoscopy is allowed for the abdominal part of the procedure. Robotic TME is not allowed, since robotic TME possibly results in different primary and secondary endpoint results compared with laparoscopic TME.

In TaTME, conversion (to either laparoscopic or open TME) is defined as interruption of transanal TME due to technical difficulties or complications during transanal dissection, requiring completion of the majority of the TME using an abdominal approach. In laparoscopic TME, conversion is defined when completion of the dissection of the mesorectum is performed through a traditional open abdominal or transanal approach. Conversion is determined by the surgeon in case of concerns about patient safety, technical difficulties and inability to complete the TME procedure adequately or associated conditions that require treatment.

### COLOR III trial quality assurance

To ensure both surgical quality and centre capability to adhere to the study protocol, including the recruitment process and data collection, the COLOR III trial Quality Assurance Protocol has been developed and will be applied before entering into the trial.

To evaluate surgical quality, a Quality Assurance Manual and a Competency Assessment Tool for technical and oncological quality for laparoscopic and TaTME within the scope of COLOR III have been developed. These will be used for surgeon selection into the trial and to measure adherence to agreed surgical quality standards during the trial. A Delphi methodology has been applied with a peer-nominated international group of expert colorectal consultants in the TaTME technique in order to develop a technical manual and operation logbook. A TaTME Competency Assessment Tool was developed based on the results of the Delphi methodology. This tool has been validated in order to ensure acceptable reliability and validity standards prior to its implementation in the pre-trial and main trial phases.

A sign-off/sign-in process is included in the COLOR III trial in order to evaluate each centre’s capability to (i) recruit and randomise patients, (ii) comply with the treatment protocol and (iii) collect required data. Centres that wish to participate in COLOR III trial will be required to recruit and randomise 5 patients following the study protocol. A pre-trial checklist will be used to measure the compliance.

If a centre is unsuccessful in completing the Trial Quality Assurance Procedure, the evidence will be reviewed by the COLOR III steering committee and more evidence will be required for the component that is unsatisfactory. Within the scope of Surgical Quality Assurance, surgeons will be required to gain more experience with the support from COLOR III expert group and re-assessed.

The data collected during this Trial Quality Assurance Phase will not contribute to the main trial.

Before the trial entry, each surgeon will be required to submit 2 unedited videos for both laparoscopic and transanal TME. Two reviewers will assess the videos independently using the Competency Assessment Tool as the pre-trial entry procedure. During the main trial period, each surgeon will be required to submit 1 unedited video for every 3 cases for both laparoscopic and TaTME. The videos will be assessed using the Competency Assessment Tool to monitor the adherence to agreed standards.

### Follow-up

**Fig. 1:**
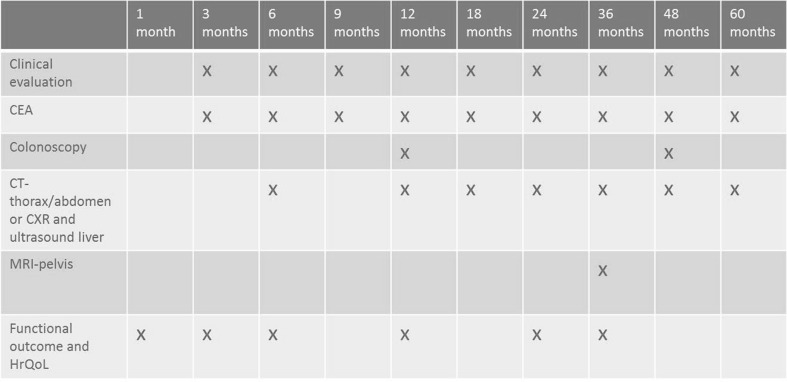
Follow-up scheme

Follow-up will be carried out (according to ESMO guidelines) yearly for a period of 5 years at the outpatient clinic (Fig. 1) [15]. More frequent visits and additional examination will be performed on indication or to the preference of the attending surgeon. Three years post-operatively, an MRI of the pelvis will be performed to exclude local recurrence. A chest radiograph and a liver ultrasound or CT scan of thorax and abdomen will be done to assess any development of distant metastases. Recurrences and deaths should be reported to the coordinating centre through the COLOR III online platform or telephone within 2 weeks of detection. Follow-up of patients with recurrent disease will be continued until at least 3 years after detection or until death. Post-operative health-related quality of life (quality-adjusted life years) and functional outcome will be evaluated at 1, 3, 6, 12, 24 and 36 months post-operatively (measured with EORTC QLQ-CR29 and C30, EQ-5D and LARS questionnaires).

### Endpoints

The primary endpoint of this trial is involvement of CRM. The CRM is deemed involved if malignant cells are found at microscopical assessment within 1 mm between the outermost part of the tumour and the CRM or between lymph nodes bearing tumour cells and the CRM. Secondary endpoints include completeness of mesorectum, residual mesorectum, morbidity and mortality, local recurrence, disease-free and overall survival, percentage of sphincter-saving procedures, functional outcome and quality of life.

### Statistical analysis

Involved CRM in laparoscopic TME for mid and low rectal carcinomas is estimated to be 7 %. The primary objective of the trial is to demonstrate a reduction in 4 % of involved CRM after TaTME compared to laparoscopic TME. To demonstrate a difference of 4 % (7–3 %) at a randomisation ratio of 2:1, 732 TaTME patients and 366 laparoscopic TME patients are required for inclusion to generate a power of 80 % for this trial. Baseline numerical data will be described in means, standard deviations or medians and interquartile ranges; baseline categorical data will be displayed in percentages. All comparative analyses will be conducted on an ‘intention to treat’ basis. Consequently, patients who are randomised to TaTME and converted to a laparoscopic or open TME will be analysed in the TaTME group. Patients who are randomised to a laparoscopic resection and converted to TaTME or open TME will be analysed in the laparoscopic group. Ninety days post-operative mortality, pathological resection margin and complication rates will be compared using the Chi-square test or an exact test if necessary. Local recurrence rate, disease-free and overall survival will be compared using the log-rank test. Exploratory analysis of the prognostic effects of various baseline factors on disease-free survival will be carried out through multivariate Cox regression. Apart from intention to treat analyses, per protocol analyses will be applied.

### Accrual and limitations

For inclusion of 1098 patients, approximately 4 years are needed. Because at the start of the trial accrual will be limited to the main centres, the estimated accrual per year will be as follows:

- Year 1: 108 patients (5 hospitals, approximately 2 patients per month)
- Year 2: 290 patients (15 hospitals, approximately 1–2 patients per month)
- Year 3: 300 patients (25 hospitals, approximately 1–2 patients per month)
- Year 4: 400 patients (30 hospitals, approximately 1–2 patients per month)

Centralising MRI review will not be limitation of this study, because MRIs will be uploaded through an online tool and can be reviewed the same day.

### Monitoring, audit and inspection

Governors will be appointed to monitor trial progress on site, as frequently as seen necessary. The medical ethical review board of the coordinating centre (VU University Medical Centre) will register the trial at the Clinical Research Bureau (CRB). The CRB will assign a data safety monitoring board (DSMB) to the trial. Interim analysis will not be performed, because the classification of this trial is not high risk. The DSMB will review the collected data and results.

### Trial registration

The trial will be registered at http://clinicaltrials.gov.

## Discussion

Worldwide, colorectal cancer is the third most common malignancy in males after prostate and lung cancer and the second most common malignancy in females after breast cancer. Each year, colorectal cancer afflicts approximately 737.000 new patients and causes about 333.000 deaths in developed countries [1]. For curative therapy, surgical intervention is required.

Rectal cancer surgery is generally considered technically more challenging than colon surgery, mainly because of the limited workspace in the small pelvis. In particular, in mid and low rectal tumours it is more difficult to achieve a radical resection because of this limited workspace and moreover due to limited visualisation. In addition, patients are confronted with high morbidity rates due to poor anastomotic techniques and conversion. Furthermore, mid and low rectal cancer surgery is associated with higher rates of permanent colostomies compared with surgery for high rectal cancer.

A quality indicator for rectal cancer surgery is the CRM. An involved CRM of 2 mm or <2 mm is associated with a local recurrence risk of 16 % compared with 5.8 % in patients without involvement of CRM (*p* < 0.0001) [16]. Various large randomised controlled trials reported an involved CRM in 7.7–16 % of patients operated because rectal cancer and higher rates of involved CRM were reported in distal rectal tumours compared to mid and proximal rectal tumours [4, 16–18].

To overcome the lack of visibility in the small pelvis and theoretically improve the rate of radical resections and decrease the rate of involved CRMs, Lacy et al. [9] introduced a transanal approach for TME in 2010. The TaTME has been developed with use of laparoscopic single port platforms to improve the quality of the TME procedure in mid and low rectal cancer. In TaTME, the tumour is distally approached through the anus with laparoscopic instruments. This facilitates a high-quality dissection of the distal mesorectum with adequate visual determination of the distal resection margin. The excellent view potentially enables nerve-sparing and sphincter-saving rectal excision.

From 2010 to date, several cohort series have been published regarding hybrid endoscopic TaTME. These series suggest that TaTME is feasible and safe regarding short-term outcomes and delivers high-quality resection specimen in selected patients. The series that excluded T4 tumours have demonstrated a promising CRM involvement of 0–5.4 % [10–13]. The largest series, including 140 patients, reported CRM involvement of 6.4 %; however, T4 tumours were not excluded and all patients with involvement of CRM were correctly predicted by MRI. Short-term morbidity and oncological results were comparable to other laparoscopic TME series [14]. A randomised controlled trial is required to evaluate the role of TaTME for rectal cancer and to assess oncological outcomes on the long term.

Before adaptation of TaTME as standard surgical therapy for mid and low rectal cancer, a well-designed study is essential to demonstrate its efficacy and safety in a multicentre randomised setting: COLOR III trial. The primary concern is oncological safety in terms of CRM involvement and local recurrence rate. Secondary concerns are safety in terms of morbidity and functional outcome. Furthermore, a major challenge in surgical cancer clinical trials is lack of consistency in surgical quality. This study aims at addressing this limitation by applying a robust surgical Quality Assurance Protocol prior to the start and throughout the clinical trial to ensure consistency and validity.
